# Alcohol: Its Place and Power

**Published:** 1858-04

**Authors:** 


### ALCOHOL; ITS PLACE AND POWER.*

A TRULY able and temperate work on a subject so prescriptively obnoxious to extreme views as alcohol is rarely met with. We welcome it gladly; and the more so when, as in the present case, it is the production of a writer who is distinguished no less as an authoritative exponent of medical science, than as an earnest and indefatigable promoter of all that can tend to the moral and social happiness of his fellow men. We are not going to discuss the many interesting points involved in the consideration of so important a subject as "the place and power of alcohol"; or to lead the reader through the mazy thread of sophisms employed by the extreme zealots on each side of this much vexed question. Such a task would demand more time and space than we can devote to it: at the same time, we think it only just, both to our author and readers, to state very concisely our own opinions on the matter at issue.

Whenever two sides of a question are diametrically opposed to one another, we think there may generally be found a *via media* between them, which includes many of the arguments in favour of both, whilst it is open to none of the objections against either. And this applies, in our opinion, to the discussion respecting the utility of alcoholic stimulants. The attempt is generally made to force upon us the alternative of total abstinence, or the evils of drunkenness. We decline, however, to accept the proffered Scylla of the teetotallers in exchange for the Charybdis of intoxication; because we feel assured that the safer and more practicable course lies between the two. Nor is this the dictate of a Laodicean policy, or of the irresolute timidity which often instigates the acceptance of a compromise, where the moral courage to adopt the right and more difficult path is wanting.

The results of experience, as well as the researches of science, show most satisfactorily that the moderate use of alcoholic drinks is one of the necessities of civilised life. In all times, and countries, and circumstances, alcohol has asserted its claims to a foremost place in the consideration of the moralist, the legislator, the social economist, and the physician, if not as indispensable to our existence, at least as ministering largely to our health, our enjoyments, and our general well being. Like many valuable agencies, its power for evil is as great, or greater

* Alcohol; its Place and Power. By JAMES MILLER, F.R.S.E., F.R.C.S.E. Glasgow: 1858.

than its power for good; but its capability of perversion no more constitutes an argument against its utilisation, than the destructive influences of fire prevent our employing it for the many purposes of necessity and comfort to which it is subservient. The question of where its usefulness ends and its hurtfulness begins, is another and very different affair; one which every man must to a certain extent determine for himself, and which no one who is honestly anxious to arrive at a truthful result will find much difficulty in settling. To any one who may feel himself embarrassed in this inquiry, not so much from want of will to make a decision at all, as from lack of knowledge upon which to frame it, we heartily recommend this little book of Professor Miller. It is most temperately written; and we are satisfied that the few points upon which we think he has rather overstated his case are more than counterbalanced by the candid, complete, and able manner in which he has treated his subject generally. If he does not always carry conviction, he never outrages reason, like most preceding writers. He has produced an eminently readable and attractive work on a most important topic, and fully deserves the thanks of society.

Though originally intended for Scotch readers, and to meet the wants of a Scotch league, the merits of this little book will secure it a ready appreciation on this side of the Tweed. Whilst it is what it professes to be, "a homely exposition of the matter, and one readily accessible to the popular hand and mind", it is at the same time possessed of qualities which will commend it to the attentive perusal of the medical practitioner, who will find in it many a truth over which he will do well seriously to ponder. As might be expected, coming from the Professor of Surgery in the University of Edinburgh, it is fully up to the present state of science, and bears the imprint of a mind which, though closely occupied by the practical duties of an arduous and responsible position, is yet well acquainted with facts and theories that are far from being "household words" in the literature of the profession. But its greatest merit is the tone of manly independence and honesty which runs throughout it, and which is of much greater importance than mere scholastic erudition, in a book that is intended to circulate largely amongst a class of readers who are frequently unable to detect the sophistry and special pleading so common to works of this kind; an honesty and fearlessness of expression, which those who have had the pleasure of listening to Professor Miller's animated discourses on this and kindred topics, will not fail to recognise as the stereotype of his living words. It is this, conjoined with the peculiar happiness of illustration which the author possesses, and the frequent brilliancy of language with which he clothes his ideas, that constitutes the great charm of the book, and will, we are sure, cause it to be very popular.

The author treats the subject of alcohol under two points of view: firstly, as to its "place" amongst the ingesta; secondly, as to its "power" upon the body when taken into the blood. We hardly think that the repetition which this arrangement involves is compensated by any gain in the matters of clearness or precision. To us, the "place" of alcohol, as a medicine, as a luxury, and as food, are so intimately connected with one another, that their divorce is impossible. In considering the "place" of a remedy in the *Materia Medica,* we must necessarily take its therapeutic "power" into account; nor can the estimation of the rank which an article of food must hold in a dietary list be a whit more dissevered from that of its effect as a tissue-forming element of the body generally. Upon logical as well as upon scientific grounds, therefore, we object to this division of the subject, into which Professor Miller has probably been led from a desire to assimilate the title of the present work to that of another which has preceded it.*

The general consideration of the properties of alcohol is premised by a brief popular explanation of the more ordinary functions of the body when in health, in which we notice that Liebig's theory of the directly calorifacient properties of the oily and saccharine constituents of food is adopted; and as the same theory crops out in other parts of the work, a few words upon it here may not be altogether amiss. The process of generalising upon facts, and of reducing a large and confused mass of evidence to a few explicit laws, is undoubtedly one of the most useful and necessary duties of the philosopher; but it is one which is very susceptible of perversion, especially in the hands of an imaginative man, or of one who as a populariser of science is anxious to bring down its more abstruse teachings to the level of the ordinary mind. This danger is well illustrated in the theory under consideration. The facts, that the majority of our economical sources of heat belong to the group of hydrocarbons, that oil is commonly burnt in lamps, and that the inhabitants of polar countries are accustomed to indulge largely in the oleaginous luxuries of the table, are considered a sufficient basis upon which to erect a theory: and it is thence assumed that the chief, if not the only, source of heat in the animal economy, is the direct oxidation of the hydrocarbon-

* Abstinence; its Place and Power. A Lecture delivered before the Young Men's Christian Association, December 30, 1856.

aceous matters taken in with the food. Now, if the latter and more exclusive statement of the theory be adopted, it is at once negatived by the fact that the chemist is acquainted with other means of supporting combustion than oxidation. Whenever chemical affinity is exhibited with much intensity, then heat is inevitably given out. We are therefore fully justified in assuming that many other chemical actions which occur in the body besides oxidation are the sources of its heat; though there can be no doubt that substantially this latter process is by far the most important.

The assertion that the direct oxidation of the oleaginous and saccharine constituents of the food is a chief source of heat, or even a source at all, is inconsistent with physiological teaching in its widest acceptation. The researches of Grove, combined with the more recent experiments of Bernard, prove that the heat of the body is derived from the general series of changes or transformations incident to nutrition, rather than from any one chemical result; and that the blood of those organs which perform their duties most energetically, and in which change of tissue goes on most rapidly, is always the warmest. It is not in the lungs, where the oxygen is absorbed, that heat is generated; for their blood is the coldest in the body: it would be as reasonable to say that the bread and meat which go to build up a mason's body also erect the edifice upon which he is employed, as to assert that the oily and amylaceous matters of the food are the only direct sources of that heat which is produced only by the disintegration of the tissues. The probable nature of the process is, that the dynamical force which is exhibited in the motion of the heart and muscles generally, in the circulation of the blood, and in the elective affinities of the tissues for the blood, is converted into its correlative force—heat; just as the dynamical force which we exert when we rub two pieces of wood together is converted into the heat produced by the friction.

The histogenetic importance of oil as an element of the food is now thoroughly recognised; and perhaps ere long we shall be able to say as much of saccharine and amylaceous matters. The researches of Bernard upon the liver have certainly done something towards settling this latter point. But the assertion that these and other hydrocarbonaceous matters serve only to sustain the animal temperature is surely unwarranted. That they do so at all, even indirectly, is only matter of inference; at any rate, we never heard that habitual gin-drinkers were a particularly warm-blooded class of people, which they certainly ought to be, according to this supposition.

We are not, however, unwilling to acknowledge that these substances are *indirect* sources of heat: but we submit that the distinction between the *direct* and the *indirect* value of calorifacients is by no means an unimportant one, as instituting some difference, theoretically, whether we shall tell a man to let a due proportion of these matters enter into the constitution of his food, in order that they may keep him warm; or whether we shall tell him to do so, in order that all his functions may be actively performed, and that, as a necessary consequence, his bodily temperature will never be deficient.

The first light in which Professor Miller considers alcohol is as a "poison"; and so long as he confines his statement to the employment of that agent in large doses, we fully accord with him. But, when he extends his assertion to the action of alcohol taken in "moderation", and condemns any denial of it as "horrible quibbling", as in the following passage, we must be excused if we part company with him.

"Some theorists, indeed, perversely argue, that though alcohol, in large doses, is doubtless poisonous, yet, because it is not apparently hurtful when taken in small quantity, or 'moderation', that therefore it is no poison. The same men may, in the same way, assoilzie every article of the animal and vegetable kingdoms that is brought as panel to their bar. Prussic acid in a full dose will kill you like a shot; but doses of two or three drops of the dilute acid, taken three or four times a day, not only do no harm, in certain circumstances, but even effect a great deal of positive good. Therefore prussic acid is no poison! And so of arsenic. Swallow an ounce, and you die in torture. But in Styria you will be told that it 'actually gives both to horses and men increased vigour, increased beauty, and an enviable rejuvenescence, when taken regularly in minute doses.' *Ergo*, 'horrible arsenic' is no true poison! Is not this horrible quibbling? Arguing thus, you virtually contend for the extinction of poisons as a class. According to your way of it, there can be no true poison. And verily there is none if alcohol be not such." (p. 26.)

It seems to us that the perverse theorists here reprobated are undeniably right. Whatever may be Professor Miller's idea of a "poison", we can form no other definition of it than as a substance which, when taken into the body, involves danger to health or life, by producing a disturbance in the functions of the body; and it is very certain that many articles of common food may act as poisons, when taken in excessive amount, which are perfectly innocuous, and even useful, in smaller quantities, *e. g.* salt, tea, mustard, etc. To take it for granted therefore, that, because alcohol acts as a poison in large doses, it also does so in smaller ones, is only begging the question; and Professor Miller, in citing prussic acid as an instance of a poison which is beneficial in small doses, is beside the mark: the truth being, that prussic acid is *not* poisonous in small doses; and therefore, when we call it a poison, we must always remember that the term is only predicated of its action in large doses. The word "poison", as Professor Miller uses it, could only be strictly applied to substances which in any quantity, however small, act prejudicially upon the organism, and are *never* attended by beneficial results: in which category alcohol certainly cannot be placed, from his own admission. Alcohol therefore is, or is not, a poison, in the sense in which most people employ the term. If it is, then common salt is equally so; and the force of the term is thereby considerably weakened. If it is not, then the advocates of temperance have one terror the less to appeal to in their denunciations of the evil effects of this agent. We leave them their choice of the horns of this dilemma.

Upon the author's remarks on alcohol as a "medicine" we have little to say: they are admirable, and show that his appreciation of its powers in that capacity is drawn from actual experience. We must, however, take exception to his quotation of the "inflaming patches" on the mucous membrane of Alexis St. Martin's stomach as the results of alcoholic indulgence. The investigations of Dr. Beaumont have shewn that this supposed inflammatory appearance is merely the natural vascular turgescence which is excited by any stimulus whatever, even by common food. And, if the case of St. Martin is to go for anything, it makes against Professor Miller in one point; for Dr. Beaumont places in his catalogue of *vitanda* tea and coffee—drinks which our author especially takes under his patronage.

From "alcohol as a medicine", we proceed to "alcohol as food": and here we can generally endorse Professor Miller's views. That alcohol is not food is very clear, if by the term food be implied only what goes directly to build up the tissues, and supply by its presence the gaps caused by their disintegration. But, if we extend our definition of food to all articles of ordinary diet that indirectly tend to nourish the body, then the claims of alcohol to rank in this latter division cannot be denied: for, if alcohol is not assimilated itself, it certainly is in many cases a powerful adjuvant to assimilation.

Under the head of "alcohol as a luxury", the author's power of writing, and his command of his subject, are well exhibited; and, as a specimen of his style, we cite the following passage, in which he deals with a very common, and, to say the least of it, a very telling objection.

"In another way I have heard the objection put. 'Beneficent Providence has filled the earth with food convenient for man's natural wants; and has clothed it too with *flowers* to regale and delight his senses. May I not look on wine in this light, and use it as I would a flower, at least occasionally?'

"The earth is fair with flowers—their fragrance is sweet, and their hues are beautiful. But even they are the better of man's hand to restrain and guide; and weeding may be done wisely, too, especially with regard to domestic interests.

"There is a place for every thing. Shut up the most fragrant flowers in a bedroom, and let the sleeper tell what he thinks of their perfume next morning. Literally, he is sick of it; and well he may be, for, through it, he is sick of all things else beside. There is belladonna—a graceful plant, with its dark luscious berries, most fair to look upon. But will you place it by the nursery window, or along the daily walk of your prattling children, who may be tempted to put forth their tiny hands and pluck the deadly poison? Nay: you will leave it where placed by Nature—in the neighbourhood of ruins, in waste places, and solitudes. And there is aconite—beautiful in its spike of deep blue helmeted flowers. Will you think it safe to put it into your garden, mayhap near a bed of the esculent horseradish, for whose root it has so often been mistaken? Better be content with some other ornament, and leave the monkshood to its indigenous mountain sides and wooded hills.

"Flowers are luxuries of the gentlest, as well as of the gayest sort. Many—nay, most—are in all respects harmless when in their proper place. Keep them there. And let those that you have nearest you, and in daily companionship, be both simple and safe; not poisonous, or even in any way hurtful, to yourselves or others.

"And so with alcohol. If you will have a luxury, take not that. Be content with some other, less formidable to you and yours.

Again:- What is fairer than the poppy, spread broadcast in the field? As Nature plants and rears it, it is a fit luxury to the eye. But let man, in his cunning device, torture the plant till it yield its juice; and *that* luxury—not for the eye, but for a grosser sense—becomes a deadly poison. So with the grape. What fairer than the vine—its climbing stem, its shady leaves, its gorgeous clusters? 'The fig tree putteth forth her green figs, and the vines with the tender grape give a goodly smell'. The ripened fruit is a 'dainty', both sweet and savoury; and the simple wine of the olden time, though not wholly without its alcoholic and intoxicating ingredient, was not unfitted to gladden the heart of man on occasions of festive mirth. But, as man's 'invention' extorted opium from the poppy, so it brought 'spirit' from the grape. Out of the simple luxuries that God gave him, wilful man has manufactured poisons. Samson's riddle is reversed—out of *meat* comes forth the *eater;* out of *sweetness* comes forth the *strong.*" (pp. 67-9.)

In the second division of the book we have the "power of alcohol" fully discussed, as a means of support in mental and bodily labour, and against heat and cold; as averting and producing disease; as an instrument of vice; and as a special opponent to moral reformation. Under some of these heads there is a slight amount of repetition of matter introduced into the former half of the book; but this was perhaps unavoidable in the division of his subject which Professor Miller has adopted. In the course of looking through this portion of the work, two or three little matters have struck us as of a rather questionable character. The old, and as we had hoped, exploded theory of spontaneous combustion is revived upon the authority of Dr. Charles Wilson. We are told that the habitual toper, "having become, as it were, a compound of an oil and spirit lamp—with a dash of phosphorus to boot—burns with a strange burning, producing little flame or heat (!) but steadily consuming away, in horrid stench, leaving but a small residue of dark and offensive unctuous dross to mark the place where he lay." Such a passage might pass current well enough in *Bleak House*, as a means for providing an easy exit for a personage who had played his part upon the stage and must be got rid of; but surely Professor Miller could not have felt the necessity of such a terror as this with which to point his moral. The drunkard has sufficiently numerous and real dangers to fear without adding to them the questionable contingency of his going off suddenly some day in smoke, 'like a snuff.'

The remarks which the author makes on the nature of the ancient wines are open to more serious objection. When he talks of the wine of the Scriptures as "weak and luscious," and containing "a small (not invariable) alcoholic ingredient;" as being "undeniably weak and fruity;" and when he says that such wines as port and sherry were unknown, he obviously forgets the many instances of the intoxicating power of wine, from the planter of the first vineyard downwards, and that the capability which wine possessed to "make glad the heart of man," must have depended upon the same properties in the earliest times, as in times present.

We had intended to say a little upon the theory of the present day, which regards alcohol as being oxydated at the expense of the tissues, and which Professor Miller seems to accept; a theory which, in our estimation, requires much more evidence to substantiate it than we at present possess. We cannot, however, help remarking, that Professor Miller seems somewhat inconsistent in his application of it. He makes the objection to alcohol, that under its employment the amount of the excreta is diminished, and that the waste matters are retained in the blood, whilst the disintegration of the tissues themselves is not only not retarded, but absolutely increased. Yet when he comes, a little further on, to eulogise his pet stimulants, tea and coffee, he asserts that they will enable a man to do as much work, and show as little apparent waste, as alcohol will; whilst, unlike it, they do not at the same time prejudicially affect the economy. Now, to use a common phrase, "what is sauce for the goose is sauce for the gander;" if alcohol act injuriously by shutting up the excreting organs, then tea and coffee, if they affect these organs in the same manner, which according to Böcker they certainly do, must be liable to the same objection. The experiments which have been made on these and other accessory articles of diet, to us appear to have been of a too limited nature to allow of our drawing any definite conclusions from them. One thing, however, is very clear, that we cannot have vital actions going on in the body without their being followed by a necessary result of waste of tissue, and consequently of elimination of excreta. Whatever royal roads may be supposed to exist to intellectual operations, there are none to bodily labour. Whatever is bought must be paid for. When a man uses any of his organs actively, he must consume an increased amount of their tissues: and when he consumes his tissues he must get rid of the results of their disintegration somehow or other; if not by his kidneys, by his lungs; and if not by his lungs, by his bowels or skin. That they should remain pent up to any extent in the blood under the use of alcohol, tea, coffee, or any other ordinary article of food, is quite inconsistent with what we know of the serious effects of the retained excreta of the liver and kidney upon that fluid. In such a case a very moderate amount of these drinks would produce most serious consequences to the system, whereas, we know, that they are often taken in large quantities for a considerable time without any prejudice.

In taking leave of our author, we must express the hope, that our having made exception to some points in his work will be taken in good part. We consider his book a very valuable contribution to our literature. The task which Professor Miller has undertaken is a useful and a noble one: we wish him God speed in it with all our heart. Far be it from us to stay his hand in anywise in his onslaught against vicious and unnecessary indulgences. But in his anxiety to convert mankind to virtuous courses, we would have him remember the saying of Montaigne: "Nous pouvons saisir la vertu de façon qu'elle en deviendra vicieuse, si nous l'embrassons d'un desir trop aspre et violent."

